# PLOD3 promotes lung metastasis via regulation of STAT3

**DOI:** 10.1038/s41419-018-1186-5

**Published:** 2018-11-15

**Authors:** Jeong-Hwa Baek, Hong Shik Yun, Gyoo Taik Kwon, Ju-Young Kim, Chang-Woo Lee, Jie-Young Song, Hong-Duck Um, Chang-Mo Kang, Jong Kuk Park, Jae-Sung Kim, Eun Ho Kim, Sang-Gu Hwang

**Affiliations:** 1 Division of Radiation Biomedical Research, Korea Institute of Radiological and Medical Sciences, Seoul, 01812 Korea; 2 Department of Molecular Cell Biology, Sungkyunkwan University School of Medicine, Suwon, 440-746 Korea

## Abstract

Procollagen-lysine, 2-oxoglutarate 5-dioxygenase (PLOD3), a membrane-bound homodimeric enzyme, hydroxylates lysyl residues in collagen-like peptides; however, its role in lung cancer is unknown. This study aimed to investigate the role of PLOD3 as a pro-metastatic factor and to elucidate the underlying mechanism. First, we experimentally confirmed the release of PLOD3 in circulation in animal models, rendering it a potential serum biomarker for lung cancer in humans. Thereafter, we investigated the effects of PLOD3 overexpression and downregulation on cancer cell invasion and migration in vitro and in vivo, using human lung cancer cell lines and a mouse tumor xenograft model, respectively. Further, PLOD3 levels were determined in lung tissue samples from lung cancer patients. Functional analyses revealed that PLOD3 interacts with STAT3, thereby expressing matrix metalloproteinases (MMP-2 and MMP-9) and with urokinase plasminogen activator (uPA) to enhance tumor metastasis. PLOD3 and the STAT3 pathway were significantly correlated in the metastatic foci of lung cancer patients; PLOD3–STAT3 levels were highly correlated with a poor prognosis. These results indicate that PLOD3 promotes lung cancer metastasis in a RAS-MAP kinase pathway-independent manner. Therefore, secreted PLOD3 serves as a potent inducer of lung cancer metastasis and a potential therapeutic target to enhance survival in lung cancer.

## Introduction

The incidence of lung cancer, one of the most common malignancies, has increased worldwide^1,2^. Despite remarkable advancements in targeted therapy for lung cancer patients, survival rates remain unchanged. More than 79% of lung cancer patients develop metastases, and the 5-year survival rate of patients with distant metastases is <5%^3^. Therefore, metastases account for increased mortality rates in lung cancer^4,5^. Despite advancements in early detection and improvements in treatment, the long-term survival of lung cancer patients remains poor. Thus, it is important to further the current understanding of the molecular underpinnings of metastatic progression in lung cancer and to apply this understanding in developing improved therapeutic alternatives. Identification of biomarkers predicting the risk of recurrence and metastasis is therefore clinically important. Recently, via proteomics analyses, we identified four proteins, namely, plasminogen activator inhibitor type-2 (PAI-2), NODAL modulator 2 (NOMO2), kinesin light chain 4 (KLC4), and PLOD3 to identify radioresistance-related genes. As these proteins have not been studied in detail^6^, it is important to investigate their role in lung cancer.

Collagens constitute a highly specialized family of extracellular matrix proteins, which maintain tissue architecture and regulate cellular responses^7–9^. Collagen production and deposition are regulated by various enzymes, including prolyl 4-hydroxylases (P4Hs), procollagen-lysine, 2-oxoglutarate 5-dioxygenases (PLODs), and lysyl oxidase (LOXs). PLOD proteins, involved in fibrosis and tissue remodeling, are also known as lysyl hydroxylases (LHs)^10,11^. Three PLOD isoforms have been characterized thus far: PLOD1 (LH1), PLOD2 (LH2), and PLOD3 (LH3)^12^. PLOD3 is a multifunctional enzyme with lysyl hydroxylase, collagen galactosyltransferase, and glucosyltransferase activity^13^, generating-specific glucosylgalactosylhydroxylysine (Glc-Gal-Hyl) residues in the Y position of X-Y-Gly triplets of collagens^14^. Lysine residues are modified in the endoplasmic reticulum (ER) lumen^15^. In addition to its ER localization, LH3 is present in the extracellular space of tissues and in serum^16^. PLOD2 specifically hydroxylates lysine residues in the telopeptide of procollagens, whereas PLOD1 hydroxylates lysine residues in the α-helical or central domain^17^. However, substrate specificity of PLOD3 is unknown^18–21^. Moreover, the extracellular function of PLOD3 is unclear, although glycosyltransferase activity of PLOD3 in the extracellular space is reportedly important for cell growth and viability. Although, the role of PLODs in cancer is unclear, their significance in other diseases has been reported. Recent studies have reported the role of PLODs in fibrotic diseases; lysine hydroxylation is impaired in Bruck syndrome^20^ and Ehlers–Danlos syndrome type VIA^22^; however, it is enhanced in fibrotic diseases^23^. Furthermore, recent studies have reported that PLOD2 expression is associated with an increased mortality risk in breast cancer patients^24^ and its inhibition suppresses metastases in sarcomas^25^. These results suggest that collagen-modifying enzymes, such as PLODs, may play critical roles in cancer cell metastasis. However, the physiological role of PLOD3 in lung cancer remains unknown. Hence, it is important to understand whether PLOD3 plays a role in lung cancer metastasis and its underlying mechanism.

Signal transducer and activator of transcription 3 (STAT3) belong to the family of intracellular transcription factors that mediate numerous physiological and pathological processes, such as cell proliferation, differentiation, apoptosis, and metastasis^26^. Enhanced STAT3 activity, reported in various human tumors, promotes cancer cell survival via therapeutic resistance^27^. Cytokines and growth factors promote STAT3 phosphorylation via receptor-associated Janus kinases, resulting in homo- or hetero-dimer formation and STAT3 translocation to the nucleus, where it functions as a transcriptional activator^28^. In response to ligand-receptor binding (interferons, epidermal growth factor, and interleukins), STAT3 is activated via phosphorylation at residues Tyr705 and Ser727 and it transduces signals from various signaling pathways by transactivating target genes involved in metastasis and proliferation^29,30^. STAT3 activation reportedly promotes cancer metastasis via matrix metalloproteinase (MMP)-2 and MMP-9, which are involved in cell migration and invasion^31,32^ owing to degradation of the basement membrane and the extracellular matrix (ECM)^33^. The serine protease urokinase plasminogen activator (uPA) also regulates cell migration via degradation of ECM components^34^. Moreover, uPA plays an important role in the maturation of other proteins, including MMPs and growth factors, via conversion of plasminogen to plasmin^35^.

Hence, the present study aimed to test the hypothesis that targeting of STAT3 enables cancer suppression at multiple levels via attenuation of the oncogenic potential of PLOD3 owing to upstream and downstream aberrations in cancer metastasis pathways. Our findings may underscore the importance of further elucidation of the role of PLOD3 in lung metastases via regulation of STAT3 and the associated molecular mechanisms.

## Results

### PLOD3 upregulation in lung cancer patients is associated with metastasis and poor prognosis

**Fig. 1:**
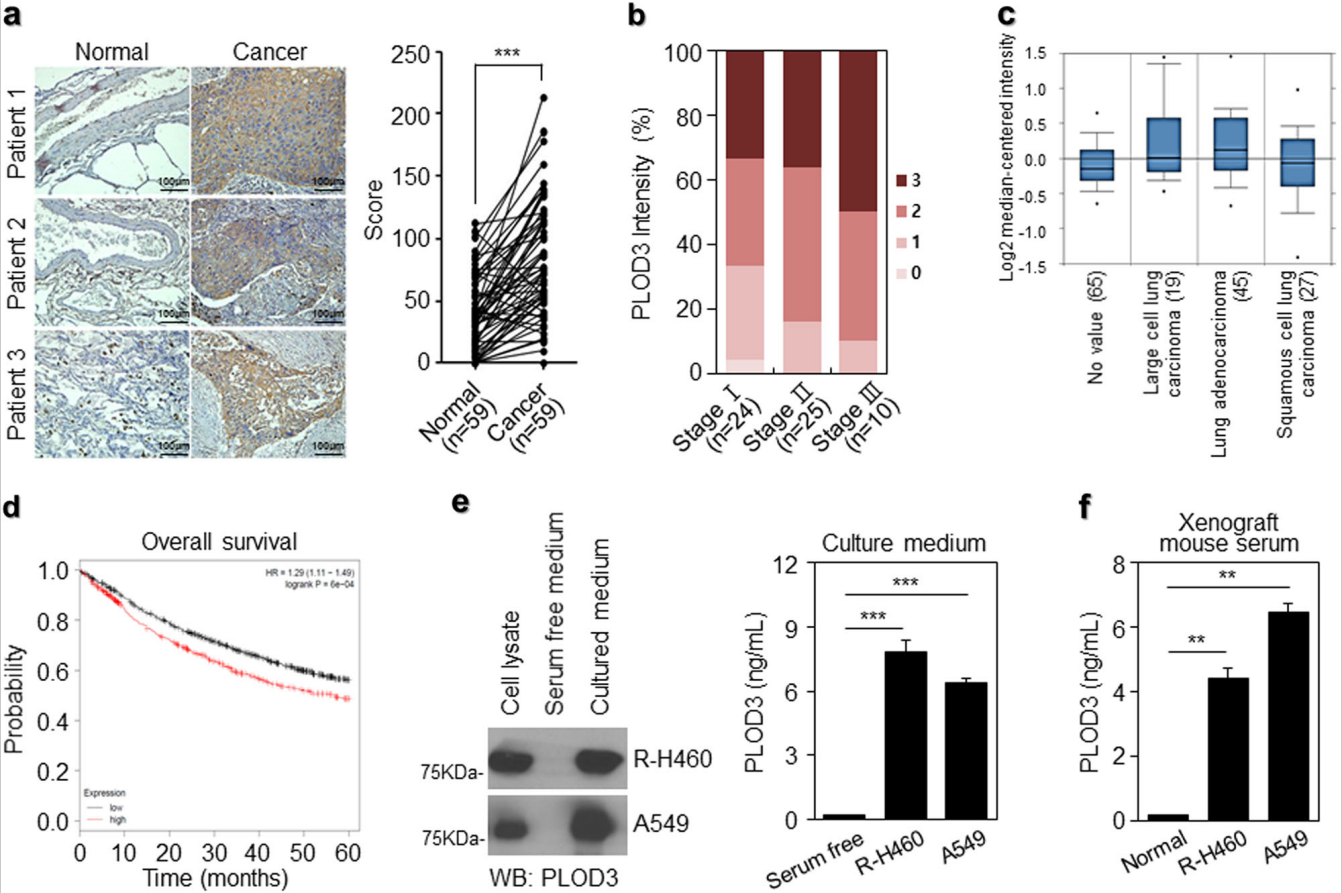
Endogenous PLOD3 plays an important role in lung cancer. **a** Representative microscopic images of lung cancers and their normal tissue counterparts stained with an anti-PLOD3 antibody (left panel, scale bar, 100 μm). Staining intensity was scored as follows in all patient samples (*n* = 59). The scores are calculated by multiplying the staining intensity with the percentage of stained cells in primary lung cancer relative to the paired normal tissue. Statistical significance was determined by Student’s *t*-test. ***P < 0.001. **b** PLOD3 upregulation is positively correlated with the stage of lung cancer in all patient samples (*n* = 59). **c** Box plot analysis of the *PLOD3* mRNA levels obtained from ONCOMINE from lung cancer patients. Gene: *PLOD3*; Analysis type: cancer vs normal analysis; Data type: mRNA; Sample type: clinical specimen; Lung. **d** The effects of PLOD3 on the overall survival of lung cancer patients, using Kaplan–Meier plotter analysis. **e** R-H460 and A549 cells were cultured up to 80% confluence, followed by changing of the medium to fresh serum-free RPMI1640. After 6 h, collected supernatants were analyzed via immunoblotting with anti-PLOD3 antibody (left panel) and enzyme-linked immunosorbent assay (ELISA) at 450 nm (right panel). Statistical significance was determined by Student’s *t*-test. ***P < 0.001. **f** R-H460 and A549 cells (1 × 10^6^ cells) were inoculated subcutaneously into the backs of nude mice. After tumorigenesis, mice were killed. Serum collected from each group was analyzed via ELISA. Statistical significance was determined by Student’s *t*-test. **P < 0.01

We first analyzed PLOD3 expression in tumor samples from lung cancer patients to determine the clinical relevance of PLOD3 expression in lung cancer. Notably, in 50 of 59 lung cancer patients, PLOD3 was upregulated in lung cancer tissue than in the corresponding normal tissue (Fig. 1a). Furthermore, we analyzed the association between PLOD3 expression and the pathological grade of lung cancer, using patient samples. PLOD3 was upregulated in pathological grade III (*n* = 10) rather than in pathological grades I (*n* = 24) and II (*n* = 25) lung cancer. Moreover, PLOD3 upregulation was significantly correlated with lung cancer stage (comparing I–II–III) (Fig. 1b). Furthermore, PLOD3 was upregulated in 37 squamous cell carcinoma patients, 15 adenocarcinoma patients, and 7 others compared to that in the corresponding tumors from the same patients (Supplementary figure 1a). To further determine the association between PLOD3 and lung cancer in clinical-pathological features, we analyzed PLOD3 expression in 156 primary lung cancer tumors relative to that in the corresponding tumors from the same patients (large cell lung carcinoma [*n* = 19], adenocarcinoma [*n* = 45], and squamous cell carcinoma [*n* = 27]) (Fig. 1c). In particular, ONCOMINE analysis data indicate that *PLOD3* mRNA was upregulated in lung cancer (Fig. 1c) and gastric cancer patients (Supplementary figure 1b). Subsequently, *PLOD3* expression levels were inversely correlated with reduced survival in lung cancer patients and in multiple human cancer types, including gastric cancers, using Kaplan–Meier plotter analysis^36,37^ (Fig. 1d, Supplementary figure 1c).

Furthermore, upon in vivo and in vitro analyses, PLOD3 was detected in the culture medium of R-H460 and A549 cells in the immunoblotting assay and sandwich ELISA (Fig. 1e). PLOD3 levels in serum-free-medium, R-H460 cell culture medium, and A549 cell culture medium were ~0.2 ng/ml, 7.8 ng/ml, and 6.4 ng/ml, respectively. We quantified PLOD3 secretion in an animal model; non-injection group (normal) and A549 and R-H460 cell groups. PLOD3 levels were quantified in serum collected after 4 weeks (Fig. 1f). PLOD3 levels in the normal group and R-H460 and A549 cell groups were ~0.19 ng/ml, 4.4 ng/ml, and 6.4 ng/ml, respectively. These results suggested that PLOD3 is secreted from lung cancer cells in vivo and in vitro, and PLOD3 is markedly correlated with a poor prognosis in lung cancer patients, playing important roles in the metastasis and lung cancer progression.

### PLOD3 promotes lung cancer cell metastasis in vitro

**Fig. 2:**
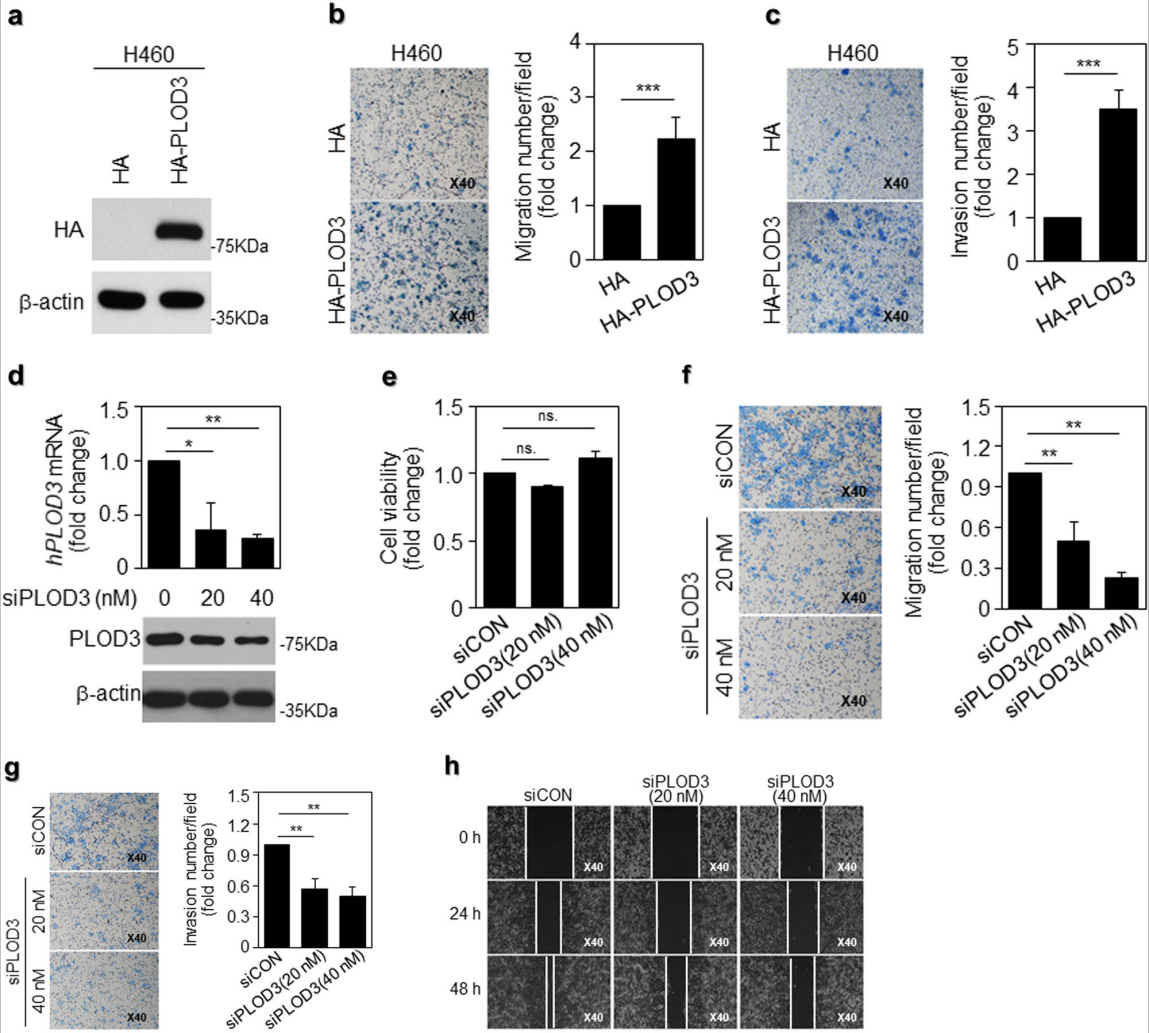
PLOD3 possesses greater potential to promote lung cancer malignancy. **a** PLOD3 protein expression was assessed via western blot analysis, following transfection of HA-PLOD3 in H460 cells for 48 h. **b** A transwell assay was performed to evaluate the motility of PLOD3-overexpressing H460 cells. PLOD3 displayed superior potential to promote cell migration. The relative numbers of migratory cells were determined and presented as the mean ± SD values from three independent experiments. Statistical significance was determined by Student’s *t*-test. ***P < 0.001. **c** The invasion assay revealed different cell motilities in PLOD3-overexpressing lung cancer cells. PLOD3 expression promoted the invasion of H460 cells. The relative numbers of invasive cells were determined and presented as the mean ± SD values from three independent experiments. Statistical significance was determined by Student’s *t*-test. ***P < 0.001. **d** PLOD3 protein and mRNA levels were determined via western blotting (lower) and quantitative reverse transcription polymerase chain reaction (qRT-PCR; upper), respectively, after the indicated concentration of siPLOD3 was transfected in R-H460 cells for 24 h. The qRT-PCR data are expressed as the mean ± SD values *P < 0.05, **P < 0.01. **e** The cell viability assay for siPLOD3-treated cells for 24 h; *x-*axis: siCON, siPLOD3 (concentration). **f** A transwell assay was conducted to evaluate the motility of PLOD3 knockdown R-H460 cells at 12 h. Statistical significance was determined by Student’s *t*-test. **P < 0.01. **g** Cell invasion assay revealed that siPLOD3 had a limited effect on pro-metastasis at 24 h in R-H460 cells. Statistical significance was determined by Student’s *t*-test. **P < 0.01. **h** R-H460 cells were treated with siPLOD3 and then incubated for 24 and 48 h. The cells were then scraped with a sterile 200-μl pipette tip for the scratch assay

To further investigate the effect of PLOD3 on lung cancer cell metastasis, we evaluated its effects on metastasis. First, to overexpress PLOD3, we transfected HA-PLOD3 into the H460 and A549 cells, which express PLOD3 lesser than R-H460 cells^6^ (Fig. 2a, Supplementary figure 2a). Exogenous PLOD3 introduction into H460 and A549 cells conferred significantly enhanced metastatic potential, as indicated by the enhanced invasive and migratory ability of these cells in a chamber assay (Figs. 2b, c, Supplementary figures 2b and 2c). To further identify the genes essential for lung cancer metastasis, we generated-specific siRNA constructs to stably knockdown *human PLOD3* (*hPLOD3*) mRNA and transfected siPLOD3 into the R-H460 cells, which express PLOD3 more than H460 cells. *hPLOD3* mRNA and PLOD3 protein were downregulated in an siRNA-dose-dependent manner (Fig. 2d). We next assessed whether PLOD3 knockdown altered proliferation in R-H460 cells. Depletion of PLOD3 in R-H460 cells did influence cell proliferation under the present experimental conditions. (Fig. 2e). However, depletion of PLOD3 greatly reduced migration and invasiveness of R-H460 cells in a siRNA-dose-dependent manner (Fig. 2f, g). Results of the wound-healing assay further supported the potent pro-metastatic effects of PLOD3 (Fig. 2h). Together, these data suggest that PLOD3 promotes metastasis in lung cancer in vitro.

### PLOD3 promotes lung cancer metastasis in vivo

**Fig. 3:**
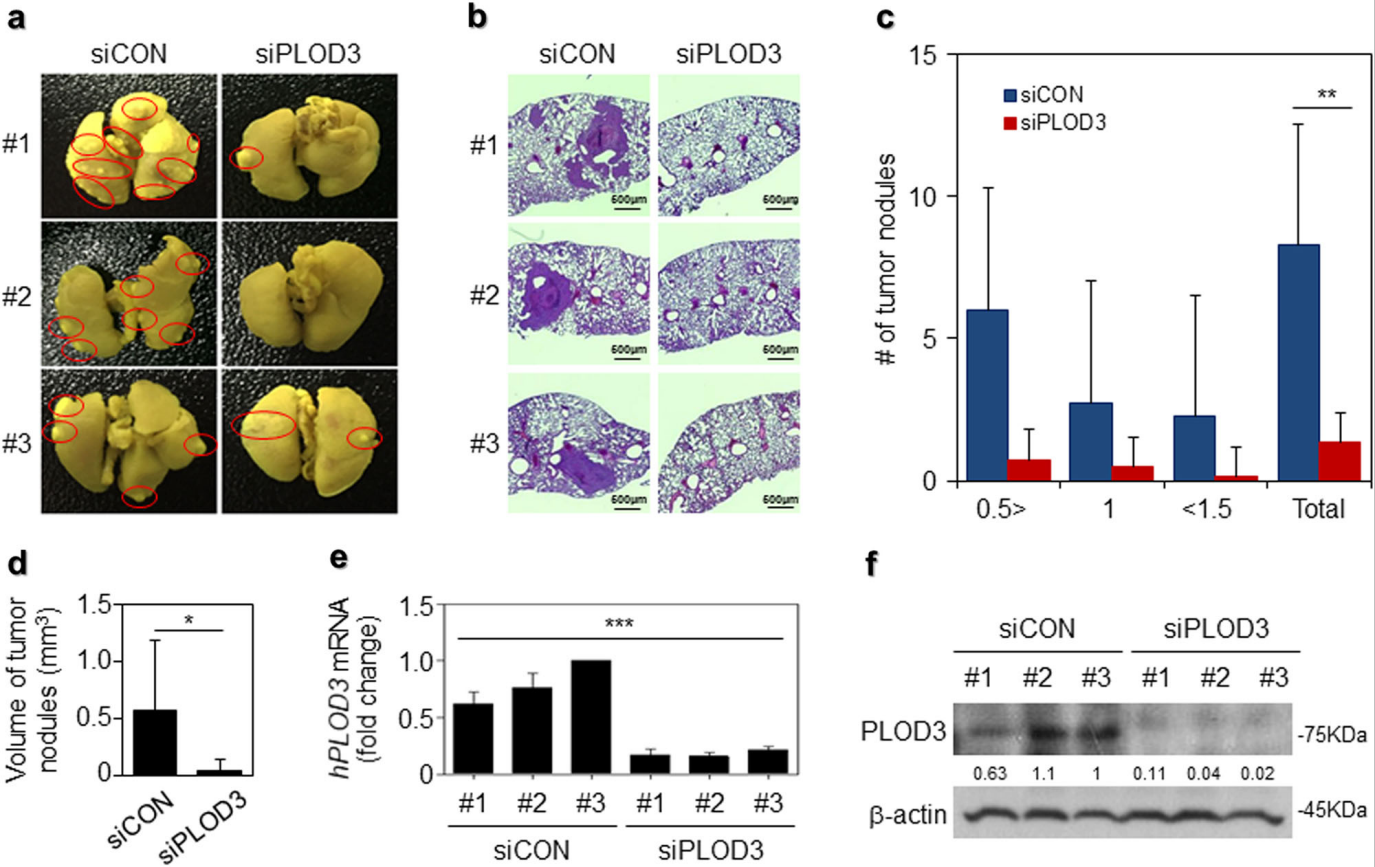
Loss of PLOD3 suppressed R-H460 tumor metastasis in vivo. **a** Representative micrographs with metastatic nodules are shown via Bouin’s staining. **b** Hematoxylin and eosin staining were performed using xenografts. **c**, **d** The number and volume of metastatic nodules were determined microscopically in PLOD3 xenograft tumors. Statistical significance was determined by Student’s *t*-test. *P < 0.05, **P < 0.01. **e**, **f** Estimation of *hPLOD3* mRNA and PLOD3 protein levels in the lung nodules. Statistical significance was determined by ANOVA analysis. ***P < 0.001

To directly evaluate the role of PLOD3 in lung metastasis in vivo, we conducted a separate analysis in nude mice via injection of control siRNA or PLOD3 siRNA in the tail vein. PLOD3 silencing reduced the incidence of lung metastasis, as determined via the nodule assay. Excised lungs after fixation in Bouin’s solution and H&E staining reduced the number of lung metastatic foci (Fig. 3a, b). PLOD3 silencing reduced the incidence of lung metastasis by decreasing the number of nodules (Fig. 3c) and tumor nodule volume (siControl (siCON)—5.8 mm^3^ vs siPLOD3—0.53 mm^3^) (Fig. 3d). Furthermore, to verify the knockdown efficiency of PLOD3, we also confirmed *hPLOD3* mRNA and PLOD3 protein downregulation in the lung nodules (Fig. 3e, f). These data indicate that PLOD3 induces metastasis in an in vivo model of lung cancer.

### Expression of MMP-2/9 and uPA during PLOD3-induced metastasis

**Fig. 4:**
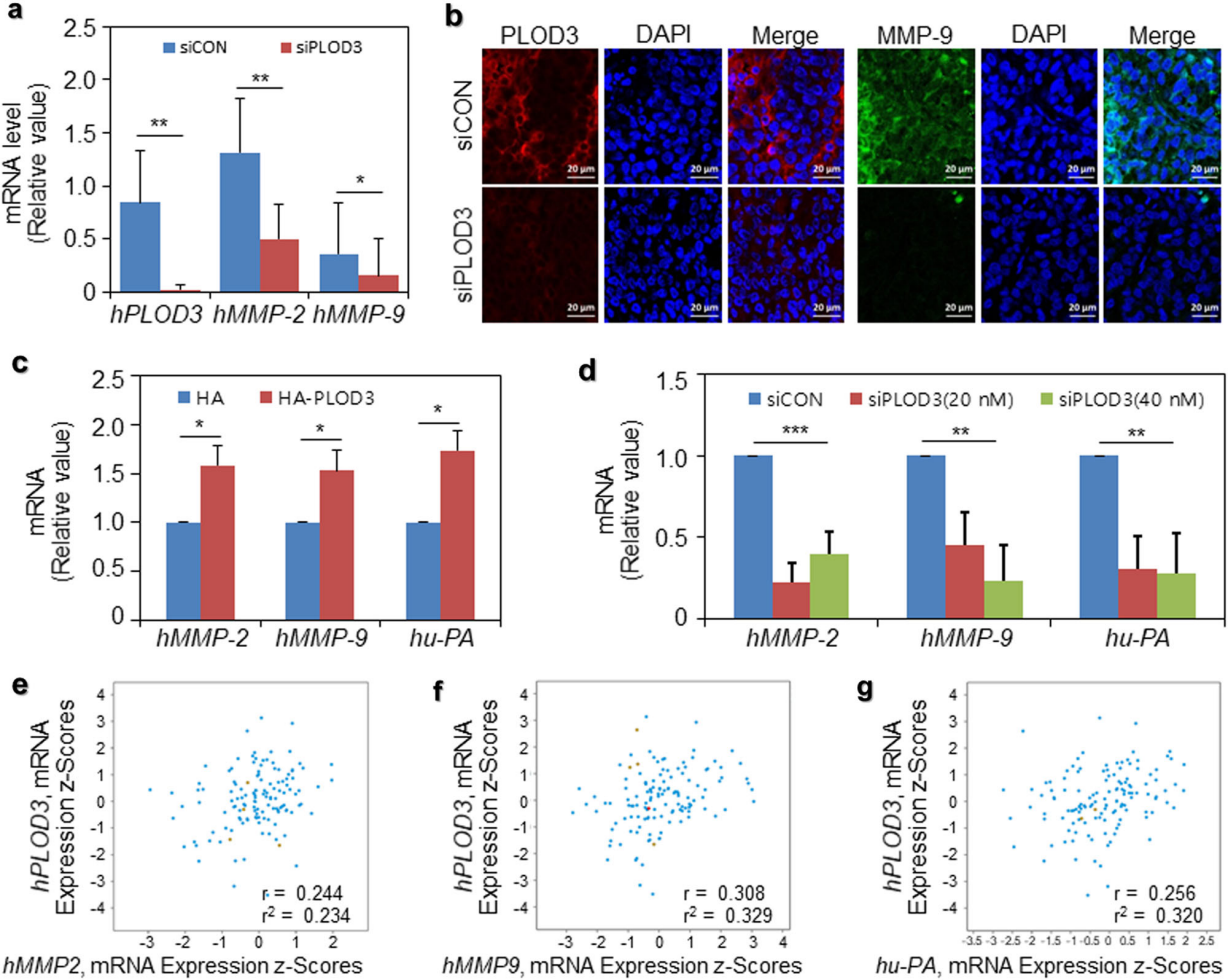
MMP-2/9 and uPA expression in siPLOD3- or HA-PLOD3-treated lung cancer cells. **a** *hMMP-2/9* mRNA levels were determined after siPLOD3 treatment in vivo. Statistical significance was determined by Student’s *t*-test. *P < 0.05; **P < 0.01. **b** In vivo immunofluorescence of siCON- and siPLOD3-treated groups. **c** *hMMP-2/9* and *hu-PA* mRNA levels after the indicated concentration of HA-PLOD3 was transfected were determined via quantitative reverse transcription polymerase chain reaction (RT-qPCR). Statistical significance was determined by Student’s *t*-test. *P < 0.05. **d** H460 cells were transfected with siCON or the indicated concentration of siPLOD3 for 24 h. *hMMP-2/9* and *hu-PA* mRNA levels were determined via RT-qPCR. Statistical significance was determined by ANOVA analysis. **P < 0.01, ***P < 0.001. **e–g** Correlation of *hPLOD3* with *hMMP-2* or *hMMP-9* or *hu-PA* transcripts in the cbioportal datasets of lung cancer patients

Further, we focused on signaling downstream of PLOD3 in relation to metastasis. Invasion during metastasis requires the upregulation of proteolytic enzymes, such as MMP-2/9 or uPA, to degrade the ECM, thereby facilitating cancer cell invasion into the surrounding tissues^25–27^. We evaluated the mRNA expression levels of metastatic genes in the R-H460 xenograft model, siCON-treated group, and siPLOD3-treated group. *Human MMP-2* (*hMMP-2*) and *human MMP-9* (*hMMP-9*) mRNA were markedly downregulated in siPLOD3-treated groups compared with those in the corresponding control groups in vivo (Fig. 4a). Furthermore, MMP-9 was downregulated in siPLOD3-treated tumors compared to the controls, upon immunofluorescence (IF) analysis (Fig. 4b). We then evaluated *hMMP-2*, *hMMP-9*, and *human u-PA* (*hu-PA*) mRNA expression levels in these two systems with HA-PLOD3-overexpressing or siPLOD3-knockdown cells, using qRT-PCR in vitro. As shown in Fig. 4c, *hMMP-2/9* and *hu-PA* mRNA were significantly upregulated in HA-PLOD3-transfected cells compared with that in control cells (Fig. 4c). Consistent with the in vivo data, the opposite effect was observed in siPLOD3-treated cells (Fig. 4d). Thus, upon analyzing the publicly available microarray datasets, we further observed a strong positive correlation among *hPLOD3* mRNA and *hMMP-2/9* mRNA, and *hu-PA* mRNA levels in lung cancer patients (Fig. 4e–g). Together, the findings from in vitro and in vivo analyses and from lung cancer patient samples demonstrate the sufficient and necessary roles of PLOD3, MMP-2/9, and uPA in promoting lung cancer metastasis.

### PLOD3 regulates STAT3 phosphorylation to promote lung cancer metastasis

**Fig. 5:**
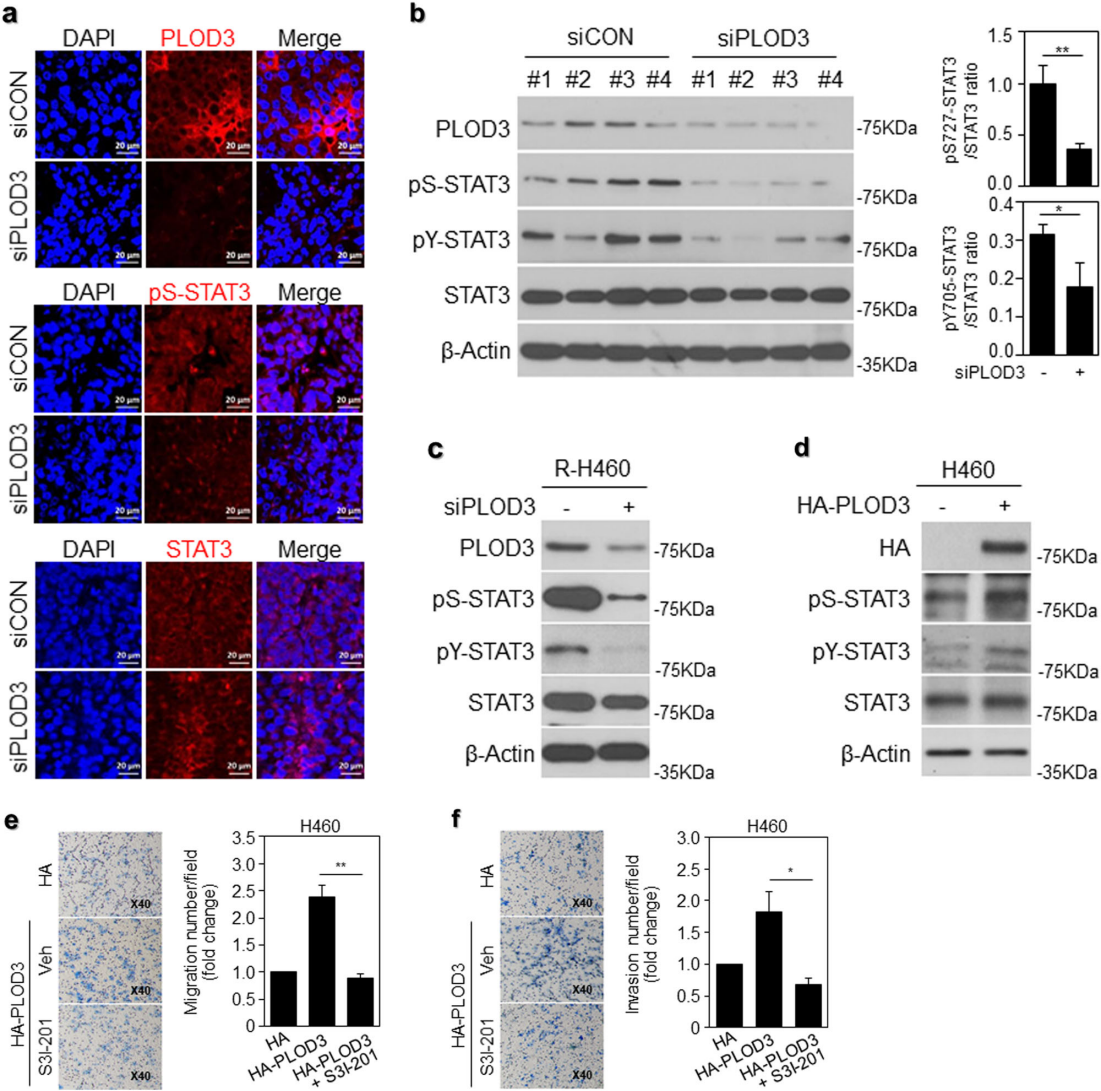
PLOD3 regulated the malignant phenotype via STAT3 signaling. **a** Immunostaining of PLOD3, pS-STAT3, and STAT3 in PLOD3 knockdown xenograft tumors. **b** Protein levels of PLOD3, pS-STAT3, pY-STAT3, and STAT3 were determined via western blotting in PLOD3 xenograft tumors. Statistical significance was determined by Student’s *t*-test. *P < 0.05, **P < 0.01. **c** Levels of the indicated proteins were determined via western blotting after siPLOD3 treatment in R-H460 cells. **d** Levels of the indicated proteins were determined via western blotting in HA-PLOD3-overexpressing H460 cells. **e**, **f** The HA-PLOD3 and STAT3 inhibitor S3I-201-treated H460 cells were analyzed via a transwell assay to evaluate cell motility and invasiveness. Statistical significance was determined by Student’s *t*-test. *P < 0.05; **P < 0.01

Considering the previously established role of STAT3 in cancer metastasis, we hypothesized that PLOD3 promotes metastasis in a STAT3-dependent manner in the model system used herein. Constitutive activation of the STAT3 signaling pathway plays a critical role in tumorigenesis and cancer progression in humans via promotion of cancer cell growth, survival, migration, and invasion^38^. Moreover, STAT3 is an important signaling mediator in malignant diseases and is persistently activated in 22–65% of non-small-cell lung cancers^39^. Therefore, we investigated whether PLOD3 knockdown inhibited the constitutive phosphorylation/activation of STAT3. As shown in Fig. 5a, in siPLOD3-treated in vivo models, constitutively activated STAT3 phosphorylation at Ser727 (pS-STAT3) was dependently inhibited, as evident from immunohistochemical analysis (Fig. 5a). Further, we quantified the in vivo data from western blotting analysis and the constitutively activated STAT3 phosphorylation of STAT3 at Tyr705 (pY-STAT3) and Ser727 was markedly decreased (Fig. 5b). In siPLOD3-treated R-H460 cells, phospho-STAT3 was significantly downregulated (Fig. 5c). In contrast, when H460 cells were treated with HA-PLOD3, phospho-STAT3 was upregulated (Fig. 5d). We confirmed that JAK activity upstream of STAT3 resulted in an overall downregulation of JAK1, whereas no effects were observed in the case of JAK2 (data not shown)^40^. In this context, these findings strongly suggest that PLOD3 knockdown effectively suppresses the constitutive activation of STAT3 in lung cancer cells. To examine the direct effect of STAT3, we used the STAT3 inhibitor, S31–201. Cells treated with this inhibitor showed decreased migration and invasion owing to PLOD3 overexpression in chamber assays (Fig. 5e, f). Our data indicate that PLOD3 regulates lung cancer metastasis directly via STAT3 signaling.

### PLOD3–STAT3 interaction is associated with progression and poor prognosis in lung cancer patients

**Fig. 6:**
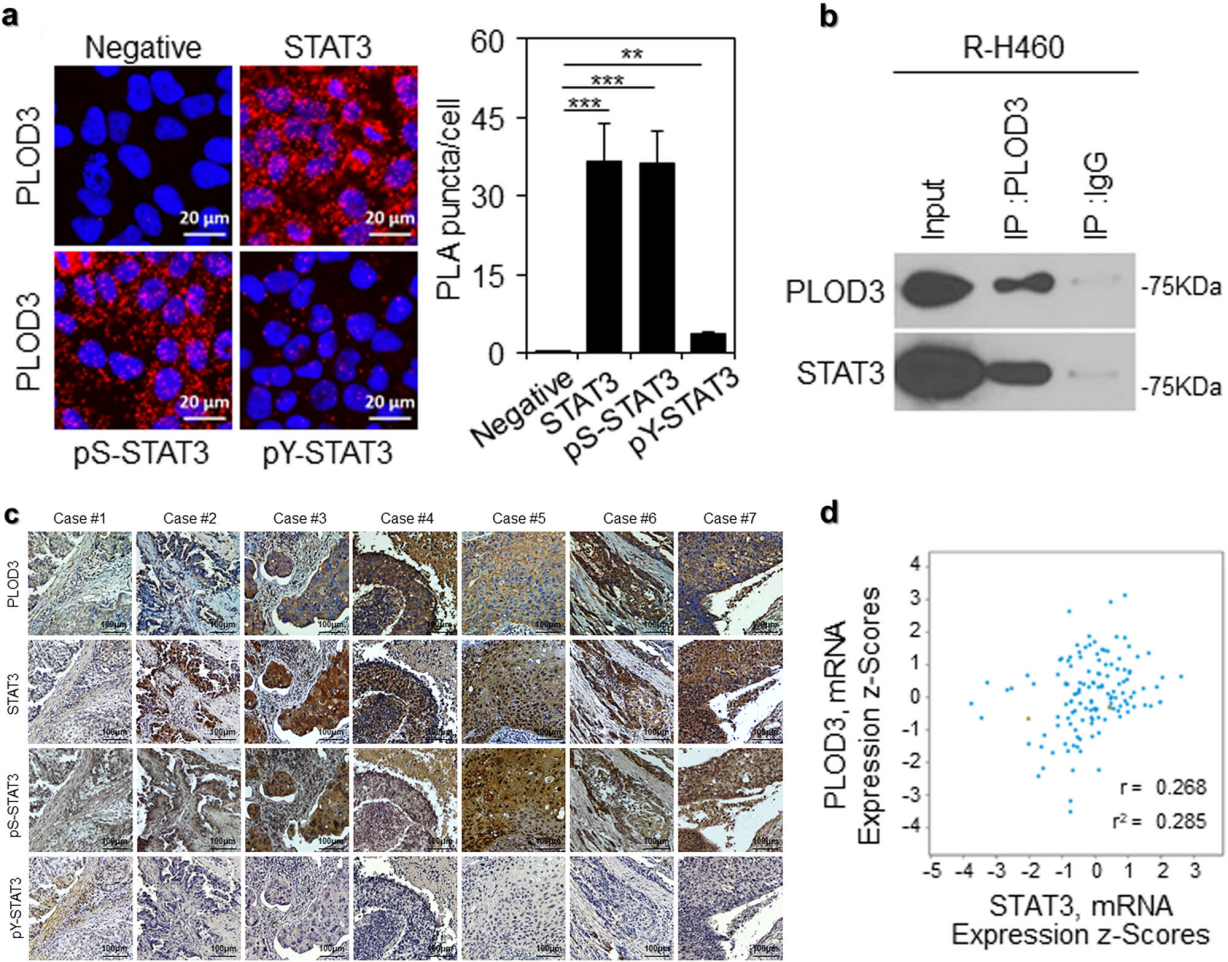
Correlation between the PLOD3-STAT3 signaling pathway and the survival and prognosis of lung cancer patients. **a** The in situ proximity ligation assay was performed for R-H460 cells stained with STAT3, pS-STAT3, and pY-STAT3. Statistical significance was determined by Student’s *t*-test. **P < 0.01; ***P < 0.001. **b** Immunoblotting analysis of lysates after immunoprecipitation from R-H460 cells with expressing PLOD3 and STAT3. **c** Representative microscopic images of specimens of lung cancer patients stained with an anti-PLOD3, anti-STAT3, anti-pS-STAT3, and anti-pY-STAT3 antibodies. **d** Correlation between PLOD3 and STAT3 transcripts in the cbioportal datasets of lung cancer patients

Importantly, the present results help elucidate the mechanism underlying PLOD3-mediated regulation of STAT3 function via direct interactions; PLOD3 may induce robust binding of STAT3 to its consensus-binding elements and upregulate target genes, thereby promoting lung cancer metastasis. To confirm this supposition, we analyzed STAT3 and PLOD3 interactions in R-H460 cells via an in situ proximity ligation assay (Fig. 6a). In particular, pS-STAT3 robustly interacted with PLOD3 compared with pY-STAT3. This was confirmed via immunoprecipitation analyses. As shown in Fig. 6b, PLOD3 directly interacted with STAT3. To determine whether PLOD3 and STAT3 coordinate to promote lung cancer metastasis in patients, we analyzed the potential correlation between PLOD3 and STAT3 expression levels in lung cancer patients. We performed immunohistochemical analysis of PLOD3, pS-STAT3, pY-STAT3, and STAT3 levels, using tumor tissue samples harvested from lung cancer patients (the same samples used in Fig. 1). The results in Fig. 6c indicate that pS-STAT3 levels were positively correlated with PLOD3 upregulation in lung cancer tissues. Furthermore, analysis of the publicly available microarray datasets revealed a correlation between PLOD3 and STAT3 mRNA levels in lung cancer patients (Fig. 6d). These data suggest that an activated PLOD3-STAT3 signaling pathway is correlated with a poor prognosis in lung cancer patients, and pS-STAT3 plays a major role in comparison with pY-STAT3 in regulating PLOD3 during metastasis.

### PLOD3 promotes lung cancer metastasis in a RAS-MAP kinase pathway-independent manner

**Fig. 7:**
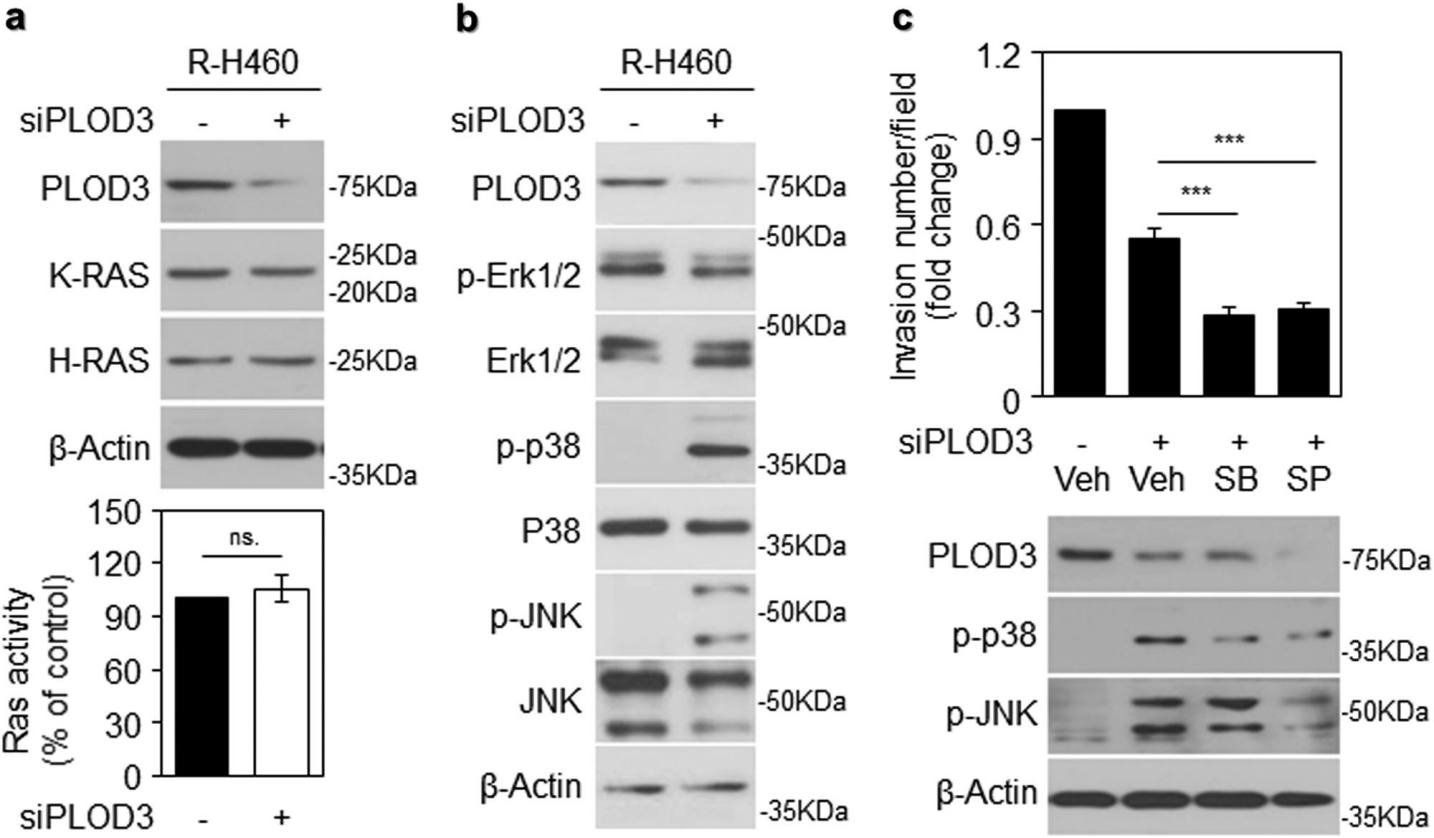
PLOD3-induced metastasis is independent of the RAS-MAPK pathway. **a** Ras expression assessed via western blot analysis following transfection with siPLOD3 in R-H460 cells (upper). Ras activity was determined via an enzyme-linked immunosorbent assay-based activity assay after siPLOD3 treatment in R-H460 cells (lower, *x*-axis: siCON, siPLOD3). **b** Protein levels of MAP kinases were determined via western blotting after siPLOD3 treatment in R-H460 cells. **c** A cell invasion assay showing that SB and SP have a limited effect on pro-metastatic PLOD3 in R-H460 cells. (upper). Protein levels of indicated proteins determined via western blotting after treatment with siPLOD3 and MAP kinase inhibitor (p38 inhibitor, SB203580 (SB); JNK inhibitor, SP600125 (SP)) in R-H460 cells (lower). Statistical significance was determined by Student’s *t*-test. ***P < 0.001

Further, to determine whether PLOD3 is required for Ras-induced metastasis, we examined K-Ras and N-Ras levels via western blotting after siPLOD3 transfection in R-H460 cells. Protein expression levels in siPLOD3-treated cells were almost similar to those in control cells (Fig. 7a, upper panel). Subsequently, upon analysis of Ras activity in siPLOD3-treated cells, Ras activity was almost similar between the two groups (Fig. 7a, lower). MAP kinase activation and STAT3 signaling upregulate MMP-2/9 during the induction of metastasis^41^. Activation levels of MAP kinase proteins (p38 and JNK) increased after siPLOD3 transfection in R-H460 cells (Fig. 7b). Accordingly, inhibitors of MAP kinase proteins were used to distinctly identify the signal transduction pathways regulated by PLOD3. The levels of invading cells decreased significantly upon SB and SP treatment in siPLOD3-transfected cells (Fig. 7c, upper). Western blotting revealed that siPLOD3-induced upregulation of p-p38 and p-JNK was attenuated upon SB and SP treatment (Fig. 7c, lower panel), indicating that PLOD3-induced metastasis is mediated independently via MAPK signal transduction pathways.

## Discussion

The morbidity and mortality of lung cancer patients predominantly results from primary tumor cell invasion and metastasis to secondary sites. In this context, we first identified that PLOD3 is one of the most widely upregulated proteins in human lung cancers and observed that PLOD3 upregulation is significantly associated with tumor progression and advanced stages of lung cancer. Furthermore, secreted PLOD3 in the blood of the animal model was identified for the first time in this study, suggesting an application of PLOD3 as a prognostic biomarker.

PLODs are implicated in metastasis because of their role in regulating collagen biosynthesis. Collagens provide the scaffold for ECM assembly and are considered “highways” for cancer cell migration^42^. Increasing evidence indicates that collagens support a barrier for migration and enhance metastasis in a collagen organization-dependent manner. Studies involving various types of human cancers have reported that components of stabilized collagen are promoted via different intra- and inter-molecular covalent cross-linkages^24,25,42^, and the different types of collagen organization are determined after cross-linking via hydroxylation of collagen telopeptidyl and helical Lys residues^43^. These changes are primarily mediated by lysyl hydroxylases, which are encoded by distinct *PLOD* genes^25^. Thus, we predicted that PLOD may be involved in cancer metastasis. Recently, Wang et al. reported that PLOD3 silencing decreases proliferation and viability in fibrosarcoma HT-1080 cells^44^. Moreover, PLOD3 overexpression has been reported in gastric, colorectal, and pancreatic cancers and glioma^45–47^. In addition, Tsai et al. and Shen et al. reported that PLOD3 overexpression promotes tumor progression and is associated with a poor prognosis in gliomas and hepatocellular carcinoma (HCC)^48,49^. These results indicate that PLOD3 may be an oncogene associated with tumor malignancy in glioma and HCC patients. These reports indicate that PLOD3 is associated with tumor progression, and *PLOD3* mRNA upregulation is associated with an unfavorable prognosis^50,51^. Furthermore, PLOD2 expression has recently been reported as a useful biomarker for the effects of antiangiogenic treatment for malignant cancers^42,43^. Furthermore, PLOD2, induced under hypoxia, is a potential novel prognostic factor for HCC following surgery^52^. Although PLOD3 has been assessed in multiple cancers, its regulation in lung cancers is unclear.

Herein, PLOD3 was first confirmed to be frequently upregulated in lung cancer patients upon immunohistochemical analysis. Clinical data also revealed that PLOD3 expression was significantly correlated with the pathological grade of lung cancer. Moreover, PLOD3 upregulation is associated with reduced overall survival in lung cancer and gastric cancer patients, and PLOD3 serves as a prognostic marker in these patients. Our finding that genetic depletion of *PLOD3* in mice was sufficient to selectively impair lung metastasis reveals an essential role in the physiological modification of PLOD3 during metastasis. The *PLOD3* siRNA model system used herein allowed for the elucidation, for the first time, of PLOD3 as a selective marker of metastasis. Unexpectedly, the *PLOD3* siRNA model system initially exhibited metastasis 12 h after siRNA transfection and apoptosis in the late period (We are currently expanding this mechanistic investigation). This is an interesting finding regarding the importance of PLOD3 upregulation, as it indicates a temporal dual function of PLOD3. Thus, interference with metastasis using siPLOD3 may represent an alternative to conventional chemotherapy for lung cancer, as most patients diagnosed with distant metastasis have a decreased survival rate.

In particular, the role of PLOD3 in promoting lung cancer metastasis has remained unclear. Initially, the correlation of PLOD3 with MMP and uPA expression was related to metastasis, upon analysis of data from a publicly available patient database. Recently, Dayer et al. reported that recruitment of MMP-9 to the fibroblast cell surface by LH3 triggers transforming growth factor-β (TGF-β) activation and fibroblast differentiation, suggesting an association between PLOD3 and MMP-9^53^. To our knowledge, the present study is the first to report that PLOD3 may inhibit lung cancer invasion by decreasing MMP-2, MMP-9, and uPA activity. However, the mechanism underlying PLOD3-mediated upregulation of MMPs and the signaling pathway by which PLOD3 regulates lung cancer cell metastasis remain unknown. Furthermore, the results from mechanistic studies, based on the observed selective regulation of lung cancer metastasis upon PLOD3 depletion, identified STAT3 as the binding partner of PLOD3. STAT3 is a constitutively upregulated and activated signaling molecule in cancer, which plays an essential role in cancer cell invasion upstream of MMPs in humans^25^. STAT3 plays an important role in metastasis in different cancer models, including lung cancer, by participating in multiple steps of metastasis^26–28^. STAT3 is extensively regulated via phosphorylation and transduces signals from various signaling pathways, including the transactivation of the target genes mediating metastasis and proliferation^28,31^. Importantly, the present results show that the mRNAs of STAT3 target genes involved in metastasis, including MMP-2 and MMP-9, were markedly downregulated upon PLOD3 depletion. Moreover, STAT3 inhibition greatly attenuated invasion by exogenous PLOD3-expressing H460 cells. These findings elucidate the mechanism underlying PLOD3-induced metastasis and indicate an indispensable role of PLOD3 in the regulation of the STAT3 pathway in this process.

These results render PLOD3 as the first upstream coactivator of STAT3 and increase the possibility that the commonly observed STAT3 upregulation in human lung cancer may be mechanistically associated with the upregulation of PLOD3 oncogene. The mechanistic study clearly revealed that PLOD3 knockdown inhibits STAT3 activation, suggesting that inactivation of STAT3 signaling by PLOD3 may serve as an effective therapeutic approach for lung cancer. The mechanism underlying the MMP upregulation and promotion of invasiveness in PLOD3-overexpressing lung cancer cells warrants further investigation. To completely elucidate the mechanisms underlying PLOD3-induced lung cancer cell migration and invasion, we further investigated the MAPK-associated signaling pathways in cellular invasion. Many studies have assessed the association between the MAPK signaling pathway and cancer metastasis and invasion^54^. However, the present study reports that activation of the Ras/MAPK signaling pathway is not essential for PLOD3 knockdown-induced invasion of lung cancer cells. Furthermore, hydroxylation of the monovalent protein can modulate protein stability, thus regulating STAT3-Y705 phosphorylation by reducing JAK1 stability upon PLOD3 inhibition. In addition, hydroxylation can modulate the protein–protein interactions, thereby regulating STAT3 phosphorylation by regulating the binding of STAT3 to its corresponding kinase^40^. Hence, it is anticipated that hydroxylation regulates STAT3 phosphorylation via STAT3 and PLOD3 in the complex by binding in the cytoplasm. In addition, PLOD3 knockdown is expected to downregulate JAK1; therefore, we consider that the JAK-PLOD3-STAT3 axis plays an important signaling role in PLOD3-induced lung cancer metastasis.

Thus far, the mechanisms underlying tumor cell migration and invasion involving PLOD3 have been unknown. EMT, characterized by downregulation of epithelial markers, particularly E-cadherin, and upregulation of mesenchymal markers, particularly vimentin, N-cadherin, and several key EMT transcription factors, e.g., snail and slug, is crucial for cancer cell migration and invasion in various cancer types^55^. Therefore, to elucidate the precise mechanisms underlying PLOD3-induced cell migration and invasion, the effects of PLOD3 on EMT-associated proteins require further investigation. Further studies are required to investigate the clinical relevance of quantifying the expression of secreted PLOD3 to develop a new target biomarker. Furthermore, PLOD3 and rapid analysis of biopsy specimens via pathological examination (picrosirius red and Masson’s trichrome staining) will provide a credible prognostic signature for the clinical evaluation of genes, such as *EGFR*, *KRAS*, and *ALK*^56,57^.

In conclusion, the present study identified PLOD3 as a novel pro-metastatic factor in lung cancer and as a novel tumor oncogene with an essential role in inducing metastasis, potentially serving as a promising prognostic biomarker for lung cancer. Additionally, verification of the serum levels of secreted PLOD3 confirmed the value of the model and the strategy for the identification of novel candidate prognostic biomarkers with clinical potential. We suggest that monitoring of serum PLOD3 levels may, in most cases, provide a more sensitive method for early detection of potentially curable cases of lung cancer metastasis.

## Materials and methods

### Cell culture and treatment

Human lung cancer cell lines (H460 and A549) were purchased from the ATCC (Manassas, VA, USA), and we generated a radioresistant H460 (R-H460) cell line derived from parental radiosensitive H460 lung cancer cells treated cumulatively with 2-Gy radiation twice a week for 20 weeks^20^. H460, A549, and R-H460 cells were cultured in RPMI1640 medium supplemented with 10% fetal bovine serum. Cells were irradiated using a ^137^Cs-ray source (Atomic Energy of Canada, Ltd.) at a dose rate of 3.81 Gy/min. Cells were treated with SB203580 or SP600125 to block MAP kinase activation or S3I-201 as a STAT3 inhibitor.

### Immunohistochemistry

Lung cancer tissue arrays (Cat No. CC5, CCN5) were obtained from Superbiochips Laboratories (Seoul, Korea). Tissue arrays contained 59 sections prepared from tumor tissue obtained from 59 lung cancer patients. Immunohistochemical staining was performed using anti-PLOD3 rabbit polyclonal antibody (Proteintech Group; 1:50), followed by the avidin–biotin–peroxidase method in accordance with the manufacturer’s instructions (Invitrogen). The results were categorized on the basis of the staining intensity: “negative” when the total score was 0 and “positive” when the total score was ≥1. The percentage of staining was analyzed using the ImageJ software (National Institutes of Health, Bethesda, MD, USA).

### Data mining using ONCOMINE database, Kaplan–Meier plotter database, and cBioportal

*PLOD3* gene expression was analyzed using microarray gene expression datasets from the ONCOMINE database (https://www.oncomine.org). The cancer type was defined as lung cancer; data type, mRNA; analysis type, cancer vs normal analysis. Kaplan–Meier survival curves relative to *PLOD3* expression were generated for lung cancer. Data were analyzed using the KM-plotter (http://kmplot.com/). Co-expression data for PLOD3 and MMP-2, MMP-9, and uPA were extracted from the cancer TCG RNA-seq data in the cBioportal database (https://www.cbioportal.org, Lung Squamous Cell Carcinoma, TCGA, Provisional).

### PLOD3 secretion assay

R-H460 or A549 cells were cultured at a density of 80%, and then the medium was changed to serum-free RPMI1640. Cells were incubated for 6 h and centrifuged at <centrifuge speed> for <duration> at <temperature>; thereafter, the supernatants were collected for the PLOD3 secretion assay via western blotting (WB) and an enzyme-linked immunosorbent assay (ELISA) (MyBioSource, Cat No. MBS2516046). Mouse blood samples were collected in a tube containing K2EDTA (BD Microtainer) and centrifuged at 3000 rpm for 30 min at 4 °C. The supernatants were collected for the PLOD3 secretion assay via ELISA.

### Western blot analysis

Western blot analyses were performed as described previously^6^, using the following primary antibodies: anti-PLOD3 (Proteintech Group, Inc., Chicago, IL, USA); anti-HA, anti-ERK1/2, anti-p38, anti-K-RAS, anti-H-RAS (Santa Cruz Biotechnology Inc.); anti-p-anti-ERK1/2, anti-p-p38, anti-p-JNK, anti-JNK, anti-STAT3, anti-p-STAT3 (Y705), and anti-p-STAT3 (S727) (Cell Signaling Technology Inc.) antibodies. β-actin (Sigma) was used as a loading control.

### Plasmid construction and transfections

Plasmids were constructed via standard recombinant cloning techniques and all changes were verified via DNA sequencing. Human PLOD3 cDNA (wild type) purchased from OriGene (Cat No. SC324563) was amplified via polymerase chain reaction (PCR) and cloned into pcDNA3.1-HA vectors. Wild-type PLOD3 was generated using primers 5ʹ-GATGGATCCATGACCTCCTCGGGGCCTGGA-3ʹ (BamHI) and 5ʹ-TGAATTCTCAGGGGTCGACAAAGGAC-3ʹ (EcoRI). Thereafter, PLOD3 was inserted between BamHI and XhoI restriction sites in pHA vectors. Transfection with these plasmids was performed using Mirus 2020 Reagent in accordance with the manufacturer's guidelines.

### PLOD3 siRNA transfections

The following human-specific siRNAs, synthesized in accordance with the manufacturer’s instructions, were used: PLOD3 siRNA, 5ʹ-GGAAGUACAAGGAUGAUGAUGACGACGA-3ʹ (IDT). Transfection with these siRNA duplexes was performed using the Lipofectamine^®^RNAiMAX Reagent in accordance with the manufacturer's instructions.

### Transwell migration and invasion assays

Transwell migration and invasion assays were conducted as described previously^6^. A 6.5-mm Transwell^®^ with 8.0-μm pore polycarbonate membrane-coated inserts was purchased from Corning (NY, USA). Cells were seeded in 100-mm dishes (1 × 10^6^ cells) and incubated for 24 h. After transfection, cells were cultured in complete medium for an additional 8 h. Cell density was adjusted to 5 × 10^5^ cells/ml to account for non-adhered cells. For the invasion assay, 5 × 10^4^ cells in 100 μl of serum-free RPMI1640 medium was seeded in the upper chamber of the insert, with 10% Matrigel on the upper chamber membrane. Thereafter, 600 μl of RPMI1640 supplemented with 0.1% BSA was added to the lower chamber, followed by incubation for 18 h. The medium and cells were then eliminated from the upper chamber using cotton swabs. The cells were fixed with 800 μl of methanol for 1 min, stained with hematoxylin for 3 min, washed thrice with distilled water, and enumerated using a microscope.

### Cell viability assay

Cells were seeded at a density of 5000 cells per well in a 96-well plate and incubated for 24 h, in accordance with the indicated experimental conditions. To quantify cell viability, an equal volume of culture media containing Cyto X^TM^ Reagent (LPS solution) was added to the cells and incubated for 4 h. Cell viability was then quantified using Multiskan EX (Thermo) at 450 nm.

### In vitro wound-healing assay

An in vitro wound-healing assay was performed as described previously^58^. Cells were seeded in 100-mm dishes (1 × 10^6^ cells) and incubated for 24 h. After transfection, cells were cultured in complete medium for an additional 8 h. Cells were cultured in a six-well plate until confluent monolayers were formed. Each cellular monolayer was then wounded or scratched with a 200-μl tip. Cells were rinsed twice with PBS and incubated with fresh media, and images were captured at 0 h, using an OLYMPUS CKX41 inverted phase-contrast microscope at ×10 magnification.

### Tumor xenograft animal model

Five-week-old male BALB/c nude mice were purchased from Orient Bio Inc. (Gapyeong, Korea), permitted to acclimate under laboratory conditions for 1 week, and provided ad libitum access to a non-purified commercial mouse diet (Superfeed Co., Wonju, Korea) and water. Thereafter, 1 × 10^6^ R-H460 cells were subcutaneously injected into nude mice aged 6 weeks. Seven days after injection of R-H460 cells, the mice were injected with control siRNA or PLOD3 siRNA (40 μg of siRNA/mouse) via the tail vein every 2 days. Ten days after injection of R-H460 cells, 6-Gy X-ray radiation was applied to the R-H460 xenograft tumors. The tumor cells were allowed to proliferate for 3 weeks. Tumor growth was evaluated by measuring the length and width with electronic calipers, and tumor volume was calculated using the following formula: Volume = (Length × Width^2^ × 3.14)/6. Three weeks later, the injected nude mice were killed, and the tumor tissues were excised, weighed, and fixed in 4% paraformaldehyde solution for further analysis. Tumor growth was evaluated on the basis of tumor volume (mean ± SD), which was plotted against time (KIRAMS 2015–0070).

### Quantitative reverse transcription-PCR (RT-qPCR) analysis

Total RNA was isolated using an RNeasy mini kit (QIAGEN). Quantitative real-time PCR was performed in triplicate, using a PIKOREAL 96 (Thermo) and SYBR Premix Ex Taq (Takara Bio, Shiga, Japan). A two-temperature thermocycling program was used, with 42 cycles of 95 °C (denaturation) and 55 °C (annealing). Target mRNA expression levels were normalized to that of *GAPDH* in the same reaction.

### In situ proximity ligation assay

The in situ proximity ligation assay (PLA) was performed as described previously^58^. Paraformaldehyde-fixed R-H460 cells were permeabilized with 0.2% Triton X-100, washed, and blocked with blocking solution (Olink Bioscience). Mouse monoclonal anti-PLOD3 antibody (Proteintech Group, Inc.) and rabbit polyclonal anti-STAT3 antibody (Cell Signaling) were used for the proximity ligation reaction. The assay was performed using the Duolink Detection Kit with a pair of nucleotide-labeled secondary antibodies (rabbit PLA probe MINUS and mouse PLA probe PLUS; Olink Bioscience) in accordance with the manufacturer’s protocol. Nuclei were stained with Hoechst obtained from the PLA reagent kit. Amplified PLA signals, represented as red fluorescent dots, were analyzed using a confocal laser-scanning microscope (Leica Microsystems, Heidelberg, Germany).

### Immunoprecipitation

R-H460 cells were transfected with the vector under the experimental conditions for 48 h. Cells were washed twice with PBS, collected, and lysed for 30 min in NP-40 buffer (50 mM Tris-HCl (pH 8), 150 mM NaCl, 1% NP-40, and protease inhibitor (×100)) containing protease and phosphatase inhibitors. After centrifugation (10 min at 15,000 × *g*) to eliminate particulate material, the supernatant was incubated with antibodies (1:100) with constant agitation at 4 °C. The immunocomplexes were precipitated with protein A-sepharose (Sigma) and analyzed via immunoblotting.

### Statistical analyses

Cell culture experiments were performed at least in triplicate. All data are expressed as mean ± standard deviation values. Statistical differences between groups were assessed using Student’s *t*-test (two-tailed) and ANOVA analysis. *P*-values were interpreted as follows; not significant (ns), *P *<* 0.05, **P *<* 0.01, and ***P *<* 0.001.

## Electronic supplementary material

SUPPLEMENTARY FIG1

SUPPLEMENTARY FIG2

Supplementary TABLE1

SUPPLEMENTARY FIG LEGEND
