# Supplementary figures and images for: PLOD3 promotes lung metastasis via regulation of STAT3
Source: Cell Death Dis. 2018 Nov 15;9(12):1138. doi: 10.1038/s41419-018-1186-5 (PMC6237925; doi:10.1038/s41419-018-1186-5)

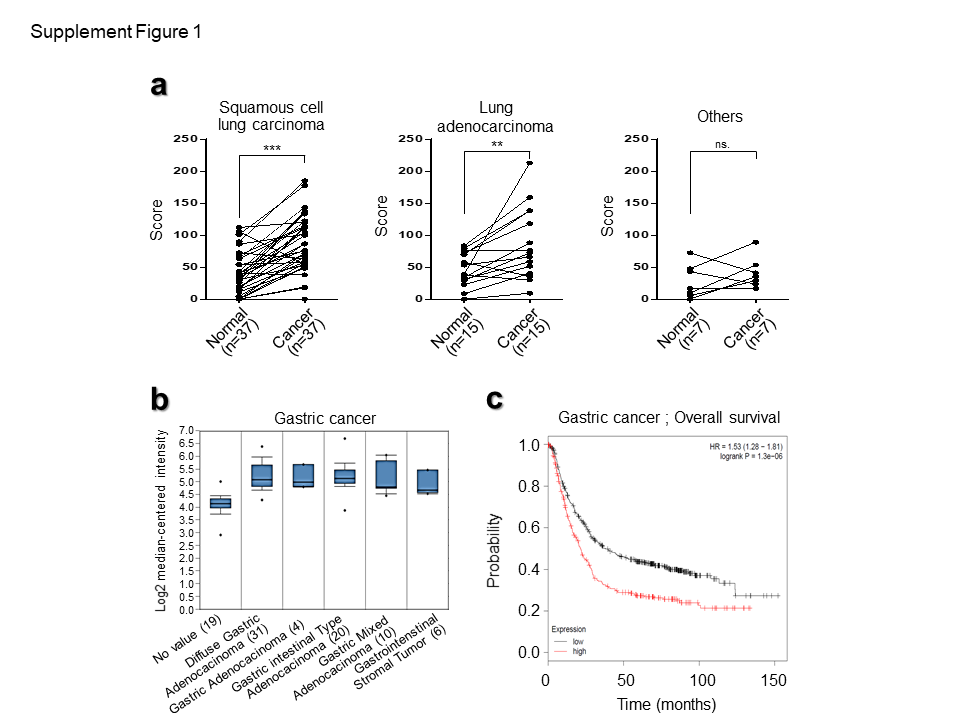

Supplement: Supplementary file 1 — SUPPLEMENTARY FIG1 [file 41419_2018_1186_MOESM1_ESM.tif]

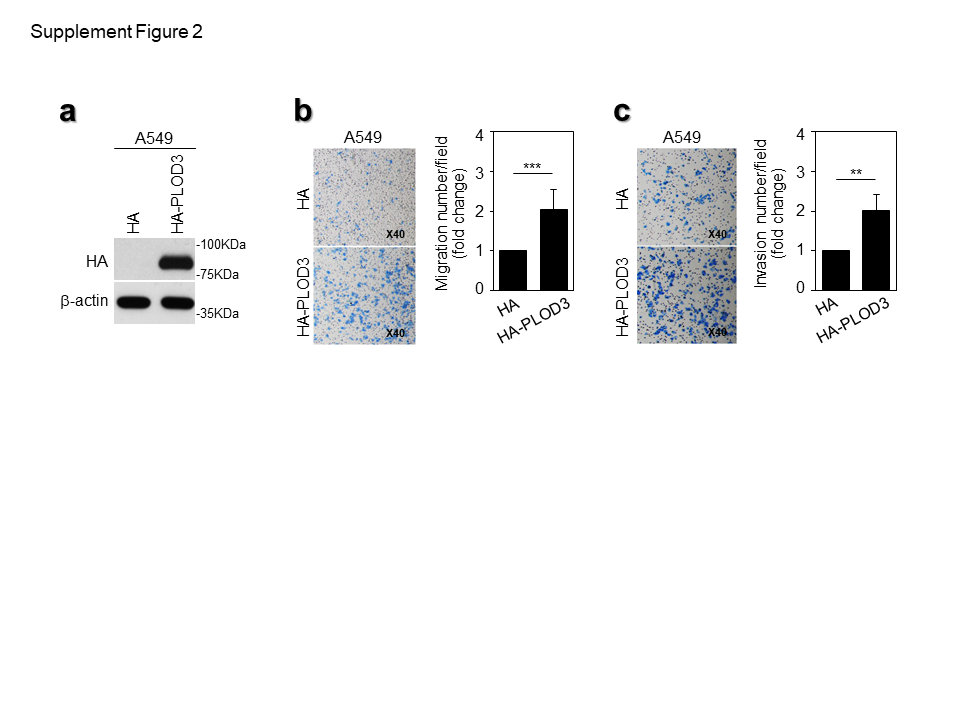

Supplement: Supplementary file 2 — SUPPLEMENTARY FIG2 [file 41419_2018_1186_MOESM2_ESM.tif]
