# Supplementary material for: PLOD3 promotes lung metastasis via regulation of STAT3
Source: Cell Death Dis. 2018 Nov 15;9(12):1138. doi: 10.1038/s41419-018-1186-5 (PMC6237925; doi:10.1038/s41419-018-1186-5)
Supplement: Supplementary file 3 — Supplementary TABLE1 [file 41419_2018_1186_MOESM3_ESM.docx]

**Supplement table 1**. List of primer sequences for qRT-PCR.

| Gene | sense/antisense | Primer sequences |
| --- | --- | --- |
| *hPLOD3* | sense | 5’-GCG CCA GTG GAA GTA CAA GGA T-3’ |
|  | antisense | 5’-CAC TTC ATC TAA AGC CCC GTT GA-3’ |
| *hMMP-2* | sense | 5’-CAA GGA CCG GTT TAT TTG GC-3’ |
|  | antisense | 5’-ATT CCC TGC GAA GAA CAC AGC-3’ |
| *hMMP-9,* | sense | 5’-GCT CTT CCC TGG AGA CCT G-3’ |
|  | antisense | 5’-TTT CGA CTT CTC CAC GCA TC-3’ |
| *hu-PA* | sense | 5’-GCC ATC TAC ACA GGA GGC ACC G-3’ |
|  | antisense | 5’-GGG TAA TCA ATG AAG CAG TGT G-3’ |
| *hGAPDH* | sense | 5’-CATC TCT GCC CCC TCT GCT GA-3’ |
|  | antisense | 5’-GGA TGA CCT TGC CCA CAG CCT-3’ |
