# Supplementary material for: PLOD3 promotes lung metastasis via regulation of STAT3
Source: Cell Death Dis. 2018 Nov 15;9(12):1138. doi: 10.1038/s41419-018-1186-5 (PMC6237925; doi:10.1038/s41419-018-1186-5)
Supplement: Supplementary file 4 — SUPPLEMENTARY FIG LEGEND [file 41419_2018_1186_MOESM4_ESM.docx]

**Supplementary** **Figure Legends**

**Supplementary Figure 1** (**a**) The expression scores of PLOD3 in tissue arrays were analyzed depending on subtype. (37 squamous cell carcinoma and 15 adenocarcinoma and 7 others ). Statistical significance was determined by Student’s *t* test. ***P* < 0.01; ****P* < 0.01. (**b**) Box plot analysis of the *PLOD3* mRNA levels in gastric cancer. Gene: PLOD3; Analysis Type: Cancer vs. Normal Analysis; Data Type: mRNA; Sample Type: Clinical Specimen; Stomach. (**c**) The effects of PLOD3 on the overall survival of gastric cancer patients.

**Supplementary Figure 2** (**a**) Expression of the PLOD3 protein by western blot analysis following transfection with HA-PLOD3 for 48 h in A549 cells. (**b**) A transwell assay was conducted to evaluate the motility of PLOD3 overexpressing A549 cells. PLOD3 stood out for its superior potential to promote cell migration. Statistical significance of cell migration was determined by a two-tailed, Student's *t* test. ****P* < 0.001. (**c**) The invasion assay showed different cell motilities in PLOD3 ectopically expressed lung cancer cells. The overexpression of PLOD3 promoted the invasion of A549 cells. The relative numbers of invasive cells were counted and presented as the mean ± SD from three independent experiments. Statistical significance was determined by Student’s *t* test. ***P* < 0.01.
